# A Metrology-Driven Self-Calibration Framework for Terrestrial Laser Scanner Sensor Systems

**DOI:** 10.3390/s26165273

**Published:** 2026-08-20

**Authors:** Honglei Yuan, Guangyun Li, Li Wang, Xiangfei Li

**Affiliations:** 1 Institute of Geospatial Information, Information Engineering University, Zhengzhou 450001, China; honglei220@163.com (H.Y.); wangli_chxy@163.com (L.W.); 2 Shanxi Institute of Surveying Mapping and Geoinformation, Taiyuan 030001, China; 15034127513@163.com

**Keywords:** terrestrial laser scanning, terrestrial LiDAR, self-calibration, calibration network design, variance inflation factor, point cloud accuracy

## Abstract

Terrestrial laser scanning (TLS), also referred to as terrestrial LiDAR, has become an essential close-range remote sensing technique for high-precision engineering surveying, deformation monitoring, industrial inspection, and cultural heritage documentation. The geometric reliability of TLS point clouds strongly depends on the effective compensation of instrumental systematic errors through in situ self-calibration. However, conventional target-based self-calibration often suffers from strong coupling between calibration parameters and exterior orientation parameters, whereas recently developed coplanarity-constrained formulations generally require highly redundant target networks, limiting their field efficiency. To address this limitation, this study proposes a variance inflation factor (VIF)-driven minimal network design strategy for efficient in situ geometric self-calibration of TLS systems. Unlike the commonly used geometric dilution of precision, VIF provides a dimensionless statistical alternative that effectively resolves the dimensional inconsistency inherent in traditional GDOP when handling mixed angular and distance parameters. A differential evolution algorithm is employed to search for hybrid calibration networks that minimize parameter coupling while preserving the physical interpretability of the National Institute of Standards and Technology (NIST) 10-parameter instrumental error model. Five digital twin simulation experiments and a physical validation experiment using a Faro Focus 350 scanner were conducted to evaluate the proposed method. The results show that the optimized network substantially reduces the number of required targets while maintaining high calibration accuracy. The final configuration, which combines VIF-optimized target placement with a dual-station height-difference constraint, reduces the condition number of the normal equations to below 60 and yields a mean system VIF close to 10. The maximum parameter correlation coefficient among the key calibration parameters is constrained to approximately 0.75, indicating near-optimal parameter decoupling under the limited field-of-view geometry of the instrument. These findings demonstrate that the proposed VIF-driven network design provides a highly effective strategy for field-efficient TLS self-calibration and improves the geometric reliability of terrestrial LiDAR point clouds in high-precision remote sensing applications.

## 1. Introduction

Terrestrial laser scanning (TLS), also referred to as terrestrial LiDAR, is a close-range active remote sensing technique capable of acquiring dense three-dimensional point clouds with high spatial resolution and millimeter-level geometric detail. Owing to these advantages, TLS has been widely used in engineering surveying, structural deformation monitoring, industrial inspection, tunnel and bridge inspection, digital preservation of cultural heritage, and urban three-dimensional modeling [1,2,3]. Furthermore, the integration of ground-based, airborne, or satellite LiDAR measurements with other geospatial methods continues to expand the necessity for robust point cloud accuracy across diverse platforms [4,5,6]. In these applications, the reliability of downstream products, including point cloud registration, surface reconstruction, displacement estimation, deformation analysis, and geometric quality assessment, is directly determined by the metric accuracy of the acquired point clouds.

Despite the rapid development of TLS hardware, instrumental systematic errors remain an important source of geometric distortion in terrestrial LiDAR point clouds. These errors may originate from the misalignment of the laser emitter and receiver, offsets between the laser beam and the trunnion axis, vertical-axis imperfections, eccentricities or tilts of rotating components, rangefinder offsets, and angle encoder errors [7,8,9]. If these systematic effects are not properly calibrated and compensated, they propagate into the observed range, azimuth angle, and zenith angle measurements, resulting in spatially correlated point cloud distortions. Therefore, rigorous geometric calibration is essential for high-precision TLS remote sensing applications [10].

In situ self-calibration has become an effective strategy for estimating TLS instrumental error parameters under realistic field conditions. Compared with laboratory calibration, field self-calibration can better account for environmental and operational factors, including temperature variation, scanner setup geometry, target distribution, and scanning configuration [11,12]. Most existing TLS self-calibration methods rely on target-based Bundle Block Adjustment (BBA), in which calibration parameters (CPs), exterior orientation parameters (EOPs), and object points (OPs) coordinates are estimated simultaneously using spherical or planar targets [13,14]. However, due to the specific spherical observation geometry of TLS, the calibration parameters are often strongly correlated with the exterior orientation parameters, resulting in ill-conditioned normal equations and physically unstable parameter estimates.

Classical TLS self-calibration using BBA is commonly formulated based on point-based collinearity equations, in which the calibration parameters of the instrument and the exterior orientation parameters of each scanning station are jointly estimated [15,16]. However, because of the specific algebraic structure of TLS spherical coordinate observations, the traditional formulation is prone to strong parameter collinearity and absorption effects [17]. In particular, several calibration parameters may exhibit high correlations with exterior orientation parameters, leading to an ill-conditioned normal equation system and reducing the physical interpretability of the estimated calibration parameters [10,18,19,20].

To fundamentally overcome the parameter coupling problem from the algebraic level, Sabzali and Pilgrim [21] proposed an innovative theory—coplanarity-constrained parametrization of collinearity equations. By introducing the scalar triple product of the baseline vector between two scanning stations and the projection rays, this method imposes epipolar geometric coplanarity constraints within the collinearity equations [22,23]. This theory significantly weakens the error compensation feedback from minor perturbations in OPs to EOPs and CPs, successfully suppressing the core parameter correlations within safe thresholds [21].

However, while this model exhibits excellent decorrelation performance, it exposes a significant practical limitation: its theoretical convergence critically depends on ultra-high data redundancy. In their published experiments, the research team deployed up to 92 precision spherical targets within the calibration room [21]. In real engineering scenarios (e.g., tunnel faces), the precise placement of even 10 spherical targets incurs substantial time costs [24]; requiring nearly a hundred targets is clearly impractical.

To address this contradiction, this study is committed to achieving minimal design of target networks through mathematical programming. Early studies (e.g., Yang et al. [25]) attempted to introduce the Geometric Dilution of Precision (GDOP) for evaluation, but GDOP suffers from dimensional inconsistency when handling TLS mixed parameters (meters for distances versus radians for angles). To this end, this paper introduces a dimensionless statistical alternative—the variance inflation factor (VIF)—for three-dimensional network optimization. This study also conducts physical origin analysis at the underlying error parameterization level, adopting the micro-physical geometric model established by the National Institute of Standards and Technology (NIST), reduced to the 10-parameter backbone model best suited to modern scanner characteristics. The Faro Focus 350 scanner [26] is used for validation. The main contributions of this paper are as follows:

1. A VIF-driven geometric network design framework for TLS self-calibration is proposed. The proposed method uses the variance inflation factor as a dimensionless measure of parameter collinearity to guide the configuration of calibration targets and scanning stations, resolving the dimensional conflicts of GDOP.
2. A minimal yet well-conditioned target network is designed for field-efficient TLS calibration. By integrating differential evolution with the coplanarity-constrained collinearity formulation, the method substantially reduces the number of required targets while maintaining the identifiability of key calibration parameters.
3. The proposed network design is coupled with a physically interpretable instrumental error model. The optimized calibration geometry is integrated with the NIST 10-parameter model, allowing the estimated parameters to be interpreted as physical systematic errors.
4. The effectiveness of the method is validated through digital twin simulations and a real TLS experiment. Five simulation scenarios and a physical validation experiment demonstrate that the proposed method improves the conditioning of the self-calibration system, reduces parameter correlation, and preserves high calibration accuracy.
5. The study provides a practical calibration network design strategy for high-precision terrestrial LiDAR applications. The proposed approach improves the field deployability of TLS self-calibration and supports reliable point cloud acquisition in engineering surveying and close-range remote sensing.

The remainder of this paper is organized as follows: Section 2 reviews the theoretical background. Section 3 rigorously derives the NIST-based physical error model and elaborates on the dimensionless VIF-driven methodology. Section 4 introduces the experimental design. Section 5 provides an analysis of simulation and physical measurement results. Finally, Section 6 concludes the paper.

## 2. Theoretical Background and State of the Art

The verification and calibration of TLS systematic errors is an interdisciplinary problem spanning geomatics engineering, precision opto-electro-mechanical instrumentation, and computer vision. Reviewing the development trajectory of this field over the past two decades, relevant research has mainly advanced alternately along two principal lines: dimensionality reduction and decoupling of adjustment mathematical models and topological optimization of field observation networks [27].

### 2.1. Traditional Self-Calibration and Limitations

In the early stages of TLS verification technology development, researchers naturally migrated the precision calibration concepts of total stations to this domain. Pioneer scholars such as Lichti [7] conducted in-depth physical analyses of systematic error sources for Amplitude Modulation Continuous Wave (AM-CW) TLS, and proposed schemes for separating collimation axis and trunnion axis errors through two-face measurements. However, most modern commercial TLS operate as highly closed ‘black boxes’ from the factory and no longer support traditional dual-face observations.

To circumvent hardware limitations, Reshetyuk [8] and others developed point-based BBA frameworks using single-face observations. Such methods establish standard single-station collinearity condition equations. Abbas et al. [15] pointed out that when the spatial scale is limited, constant-term errors and coefficient-term errors cannot generate sufficient independent gradient differences, causing extremely strong algebraic collinearity between EOPs and CPs (absolute correlation factor |ρ|>0.99), leading the normal equation matrix for adjustment computation to fall into severe ill-conditioning [15].

### 2.2. Coplanarity-Constrained Calibration Formulations

In computer vision, multi-view geometry has proven that the relative pose of cameras can be solved independently using epipolar geometry [22,28]. Sabzali and Pilgrim [21] innovatively introduced this concept into surveying-grade TLS self-calibration. Their scheme adds coplanarity conditions as strong mathematical constraints. By using the scalar triple product to strictly require that the scanning station baseline and two spatial sight lines intersect at the same point, this method integrates the originally loosely connected free network into a three-dimensional structure with rigid relative orientation [21,23]. However, to prevent the Jacobian matrix from exhibiting local rank deficiency, they relied on massive dense target deployment (>90 targets) [21]. This paper argues that the rank deficiency risk can be resolved through rigorous network topology optimization rather than blind redundant stacking.

### 2.3. Calibration Network Design and Parameter Correlation Analysis

Seeking optimal geometric configurations is a classical proposition in geodetic network design [18,27]. Lichti et al. [19] verified the decisive influence of spherical target distribution morphology on BBA output uncertainty. Early studies predominantly used GDOP theory to evaluate TLS networks. However, GDOP directly sums the traces of translation and rotation matrices, ignoring that TLS calibration parameters contain distance parameters (meters) and angle parameters (radians). The core motivation of this paper is to combine the pure statistical, dimensionless VIF with the coplanarity-constrained equation reconstruction.

## 3. Analytical Framework and Optimization Methodology

This section rigorously derives the TLS systematic error model, reconstructs the coplanarity-constrained collinearity equation framework, and elaborates the core algorithm of dimensionless VIF-driven hybrid network optimization.

### 3.1. Rigorous Physical Error Model of TLS Based on NIST

The raw spherical coordinate observations of a terrestrial laser scanner are the slope distance *R*, azimuth angle *H*, and zenith angle *V*. Their ideal theoretical mapping to the instrument’s local Cartesian coordinate system is

(1) $$\left[\begin{array}{c} X\\ Y\\ Z \end{array}\right]=\left[\begin{array}{c} R\sin V\cos H\\ R\sin V\sin H\\ R\cos V \end{array}\right]$$

Considering opto-electro-mechanical defects, actual observations contain systematic biases. Let the range error be dr, the azimuth angle error be dh, and the zenith angle error be dv; the corrected observations are then R+dr, H+dh, and V+dv.

To rigorously express the physical origins of dr, dh, and dv, this study adopts the TLS micro-physical error model led by NIST [9,29]. We reduce the classical 18-parameter model and reconstruct it into a 10-parameter core backbone model (CPs). The strict algebraic mapping is as follows:

(2) $$dr=a_{3}\sin V+a_{10}$$

(3) $$dh=\frac{a_{2}}{R\tan V}+\frac{a_{4}}{R\sin V}+\frac{a_{7}-a_{9}}{\tan V}+\frac{2a_{8}}{\sin V}+\frac{a_{1}}{R}$$

(4) $$dv=\frac{(a_{1}+a_{3})\cos V}{R}+a_{5}+a_{6}\cos V-a_{7}\sin V-\frac{a_{2}\sin V}{R}$$

The original NIST 18 parameters include azimuth/zenith circle eccentricities and second-order systematic scale errors. However, modern high-end laser scanners are generally equipped with multi-reader absolute angle encoders. This symmetric averaging underlying hardware sampling mechanism automatically eliminates the vast majority of circle eccentricities and second-order periodic errors at the physical level [17,29]. Forcibly retaining these minimally influential parameters triggers severe over-parameterization of the normal equations [15]. Strictly reducing the model to focus on 10 core physical parameters (for $a_{1}\ldots a_{10}$ parameter details, please see Table A1 in Appendix A) significantly enhances the matrix reliability of field calibration solutions.

### 3.2. Coplanarity-Constrained Collinearity Parameterization Framework

The core of this study’s adjustment is the collinearity equation under coplanarity condition constraints. To overcome the datum rank deficiency of the free network, this paper adopts a strict zero-order design (ZOD), designating the first scanning station as the absolute origin and orientation datum:

(5) $$X_{S1}=Y_{S1}=Z_{S1}=0,\;\omega_{1}=\phi_{1}=\kappa_{1}=0$$

where $\left(X_{S1},Y_{S1},Z_{S1}\right)$ represents the three-dimensional absolute translation coordinates of scanning station 1, and $\left(\omega_{1},\phi_{1},\kappa_{1}\right)$ represents its Euler angles expressing spatial attitude. By forcibly constraining these to zero, the exterior orientation elements of subsequent scanning stations are automatically transformed into relative orientation parameters.

For a spatial target *i*, while conventional collinearity equations optimize the absolute coordinates of target points, the model forcibly incorporates scalar triple product coplanarity constraints based on the translation baseline and heterogeneous direction vectors during the construction of the normal equations:

(6) $$F_{coplanar}(CPs,ROPs)=b_{j}\cdot (U_{1i}\times U_{ji})=0$$

where **CPs** represent the 10 systematic calibration parameters of the instrument; **ROPs** represent the relative orientation parameters of station *j* with respect to the datum station; $b_{j}={\left[X_{Sj},Y_{Sj},Z_{Sj}\right]}^{T}$ is the relative translation baseline vector connecting the two scanning station centers; $U_{1i}$ and $U_{ji}$, respectively, represent the three-dimensional direction vectors of target *i* in station 1 and station *j*, after system error compensation in local polar coordinates and rotation to the unified datum Cartesian coordinate system. This constraint forces the spatial skew error of homonymous rays to approach zero, cutting off the algebraic compensation channel from OP perturbations to CPs through rigid epipolar geometry [21].

### 3.3. Optimal Hybrid Network Design Driven by Dimensionless VIF

Traditional GDOP design optimizes networks by minimizing the trace of translation or rotation covariance sub-blocks. However, in TLS calibration models, the dimensions of CPs include meters and radians. Forcibly summing variance elements with different dimensions (meters and radians) is physically nonsensical and mathematically highly unstable.

To thoroughly resolve this problem, this paper introduces VIF as the core evaluation index. Let J be the m×10 Jacobian design matrix, and P be the m×m diagonal weight matrix of the observations, where *m* is the number of observations. The 10×10 normal equation matrix $N=J^{T}PJ$ and its cofactor inverse matrix $Q=N^{-1}$ are constructed. For the *k*-th parameter, its variance inflation factor ${\mathrm{VIF}}_{k}$ is strictly defined as the product of the main diagonal element of the normal equation matrix and the main diagonal element of the cofactor matrix:

(7) $${\mathrm{VIF}}_{k}=N_{kk}\times Q_{kk}$$

The algebraic essence of this formula is to compare the actual estimated variance of the *k*-th parameter with its ideal variance under complete orthogonality with other parameters, subtly eliminating the dimensional influence of each parameter itself.

To obtain the minimal network with the strongest geometric tension and thorough decoupling, this paper deeply integrates the differential evolution (DE) algorithm with the VIF evaluation engine. While other metaheuristics like Genetic Algorithms (GAs) or Particle Swarm Optimization (PSO) could theoretically be applied, DE was selected for its robust handling of continuous 3D spatial boundaries [30]. The fitness objective functional is constructed as

(8) $$f(T)=\lambda_{1}\cdot \max(\mathrm{VIF})+\lambda_{2}\cdot \mathrm{mean}(\mathrm{VIF})+\mu \cdot p_{sep}(T)+\eta \cdot p_{vis}(T)$$

The rigorous meanings of each parameter and component are as follows:

- $T=[(X_{1},Y_{1},Z_{1}),\ldots ,(X_{n},Y_{n},Z_{n})]$ is the combined continuous search vector containing the three-dimensional spatial coordinates of *n* spherical targets.
- $\lambda_{1},\lambda_{2}$ are VIF objective weight coefficients (e.g., set $\lambda_{1}=0.4,\lambda_{2}=0.6$), used to dynamically balance the suppression of local worst-case decoupling (Max VIF) and the optimization of global variance homogeneity (Mean VIF).
- μ,η is an extremely high-value penalty term coefficient, used to transform soft constraints into hard constraint boundaries.
- $p_{sep}(T)$ is the spatial anti-collision repulsion penalty function. Considering that in real engineering calibration fields target spheres must be placed on tripods or bases, it stipulates that when the distance between any two spherical targets is smaller than the specified threshold, an exponentially growing penalty value is triggered to ensure absolute feasibility of physical placement.
- $p_{vis}(T)$ is the line-of-sight visibility penalty function, used to automatically judge and eliminate invalid points occluded by scanner tripod bases or indoor columns.

This functional forces the differential algorithm to actively search for extreme geometric feature nodes within the spatial bounding box, extracting maximum relative orientation tensors with the minimum number of targets.

## 4. Experimental Design and Validation Methodology

To comprehensively and objectively demonstrate the effectiveness of the proposed algorithm, the research architecture includes systematic validation via a high-fidelity digital twin simulation platform, as well as closed-loop testing in a physical calibration field using real hardware.

### 4.1. Experimental Setup

To provide an absolutely objective evaluation benchmark, this study first constructs a high-fidelity digital twin simulation platform. The platform simulates a standard indoor space with dimensions of 7 m×8 m×4 m. Following recent advancements in Monte Carlo distortion simulation for LiDAR [31,32], we inject objective NIST 10-parameter true values into the kinematic model, and strictly superimpose Gaussian white noise onto ideal spherical coordinates according to the sensor hardware parameters of modern commercial high-precision TLS ($\sigma_{r}=2\;\mathrm{mm}$, $\sigma_{V}=\sigma_{H}=30^{\prime \prime }$). All network solution processes uniformly rely on the trust-region-driven Levenberg–Marquardt (LM) nonlinear optimization algorithm, with the residual convergence iteration tolerance extremely strictly set to $10^{-12}$, ensuring that model convergence accuracy is not disturbed by numerical truncation errors.

### 4.2. Simulation Scenarios

This paper designs the following five progressive simulation experiments from shallow to deep:

- Exp 1: Traditional single-station collinearity (absolute control points known). 16 random wall-mounted spherical targets.
- Exp 2: Sabzali scheme reproduction (90 points with dense target distribution, equielevational dual-station scanning, and coplanarity-constrained adjustments).
- Exp 3: Sabzali scheme dimensionality reduction (16 points with random sparse distribution, equielevational dual-station scanning, and coplanarity-constrained adjustments).
- Exp 4: VIF-optimized network (16 points with equielevational dual-station scanning and coplanarity-constrained adjustments).
- Exp 5: Ultimate VIF scheme (16 points with dual-station height-difference scanning and coplanarity-constrained adjustments).

### 4.3. Physical Validation Field Construction Using Faro Focus 350

#### 4.3.1. Instrument Performance and Experimental Environment

The experiments use the internationally mainstream phase-based 3D laser scanner Faro Focus 350 (nominal accuracy: range error 1 mm at 10 m, angle noise 19″) (FARO Technologies, Inc., Lake Mary, FL, USA). The physical test site is selected in an indoor calibration room with dimensions of 10 m×8 m×3.5 m, where natural unmodeled noise such as chassis micro-vibrations, ambient temperature drift, and uneven target surface diffuse reflection exists.

#### 4.3.2. Design Concept and Implementation Details

The experiment involved 4 panoramic scans per station, executed under standard stable indoor environmental conditions to mitigate external thermal or vibrational drift. The experiment adopts 18 target objects, strictly following the three-dimensional absolute coordinates output in advance by the VIF algorithm. The instrument performs panoramic scans at high resolution from two stations, and artificial height-difference intervention is imposed, forcibly introducing a setup height difference of ΔZ=1.0 m to thoroughly break the spatial symmetry hazard in the gravity direction.

### 4.4. Statistical Evaluation Metrics

To comprehensively and rigorously quantify the geometric tension and adjustment quality of the minimal network, this paper constructs a comprehensive evaluation system containing 5 core metrics based on the least squares normal equations and cofactor matrix:

1. Maximum parameter correlation coefficient (max|ρ|)

Extracts the maximum absolute value of Pearson’s correlation coefficients calculated from off-diagonal elements of the cofactor matrix. |ρ|>0.9 indicates severe algebraic absorption, while |ρ|<0.75 marks near-optimal parameter decoupling.

2. Condition number of the normal equation matrix (denoted as *k*)

The ratio of the maximum to minimum eigenvalue of the normal equation matrix reflects the numerical stability of the least-squares solution. Larger *k* values indicate more ill-conditioned system equations, highly susceptible to observation noise interference; when *k* is compressed to the $10^{1}\sim 10^{2}$ order of magnitude, it represents the adjustment matrix possessing extremely strong anti-interference capability (well-conditioned).

3. Variance Inflation Factor (Mean VIF)

Evaluates the global multicollinearity state of the entire adjustment network. In TLS minimal target networks, if the system Mean VIF can be forcibly suppressed and converged to around 10, it indicates that the network geometric configuration has approached perfect decoupling under zero-redundancy conditions.

4. Pure mathematical vector deviation (Raw CPs RMS)

Directly calculates the root mean square error between the calibration parameters inverted by adjustment and the injected true values. The pure absolute value of this metric intuitively reflects the algebraic precision of the algorithm’s approach to objective physical truth.

5. A posteriori variance factor $\left({\hat{\sigma }}_{0}^{2}\right)$

Used to evaluate the overall goodness of fit of the system, measuring whether the residual field after parameter compensation has degraded into pure white noise (no residual systematic error, ideal expected value approaching 1.0).

## 5. Results and Discussion

Before deeply analyzing the geometric tension of each network configuration, it is imperative to first rigorously assess the adjustment accuracy from both pure algebraic and observation domain dimensions. As shown in Table 1, in sparse networks lacking scientific network shape design (Exp 3), CPs RMS deteriorates to $3.25\times 10^{-3}$; whereas relying on VIF-optimized minimal networks (Exp 4 and Exp 5), CPs RMS is forcibly compressed to low magnitudes of $1.84\times 10^{-4}$ and $1.31\times 10^{-4}$, achieving algebraic-level convergence of pure mathematical vector deviations. Furthermore, an apparent anomaly is observed in Exp 2, where the Raw CPs RMS ($2.47\times 10^{-4}$) is numerically better than Exp 1 ($7.51\times 10^{-4}$) despite a much worse condition number. This occurs because the massive data redundancy (90 targets) forcefully averages out the random Gaussian observation noise in the least-squares fitting. However, its excessively high Mean VIF (342) and max|ρ| (0.99) indicate that the parameters remain severely coupled algebraically, rendering the model physically unstable for robust calibration.

Regarding observation domain accuracy, taking Exp 5 as an example, its adjustment inversion not only stably suppresses angle residuals dh and dv to 8.7″ and 26.4″, but the range residual dr is as low as 0.27 mm. This series of extremely excellent posterior precision data proves that the proposed VIF minimal scheme not only exhibits excellent decoupling tension, but also achieves precise compensation of instrumental physical errors under the limit condition of zero data redundancy, with comprehensive accuracy completely surpassing the massive blind stacking of 90 targets (Exp 2).

### 5.1. Geometric Limitations of Absolute Control Points and Dense Redundancy

**Figure 1:**
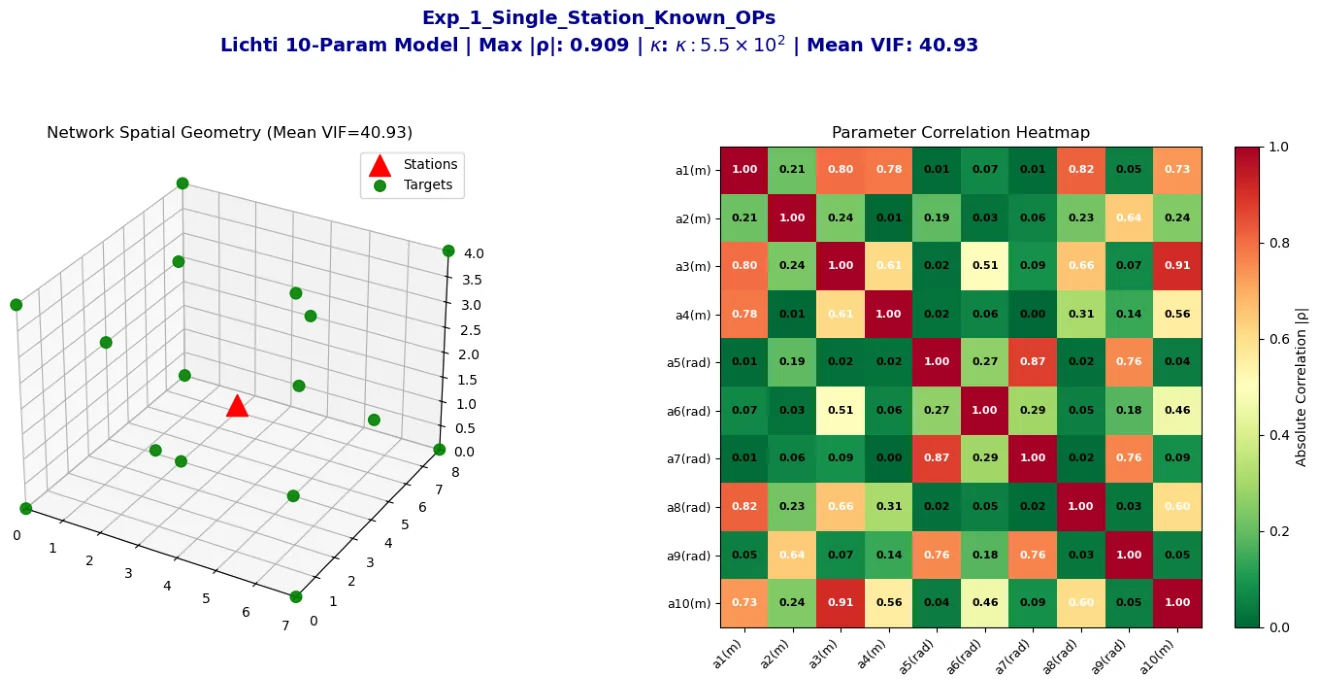
Spatial distribution of targets and parameter correlation heatmap for Exp 1 (traditional single-station absolute known coordinates with 16 points).

In Exp 1, although the system locks the absolute coordinates of 16 spherical targets, Figure 1 still shows obvious dark high-correlation regions (e.g., strong coupling between $a_{3}$ and $a_{10}$), with the maximum correlation coefficient reaching 0.91. Furthermore, the system Mean VIF reaches 41, far exceeding the well-conditioned threshold. This profoundly demonstrates that simply relying on external spatial datums cannot eliminate the underlying algebraic collinearity caused by insufficient spatial gradient variation in observation rays.

**Figure 2:**
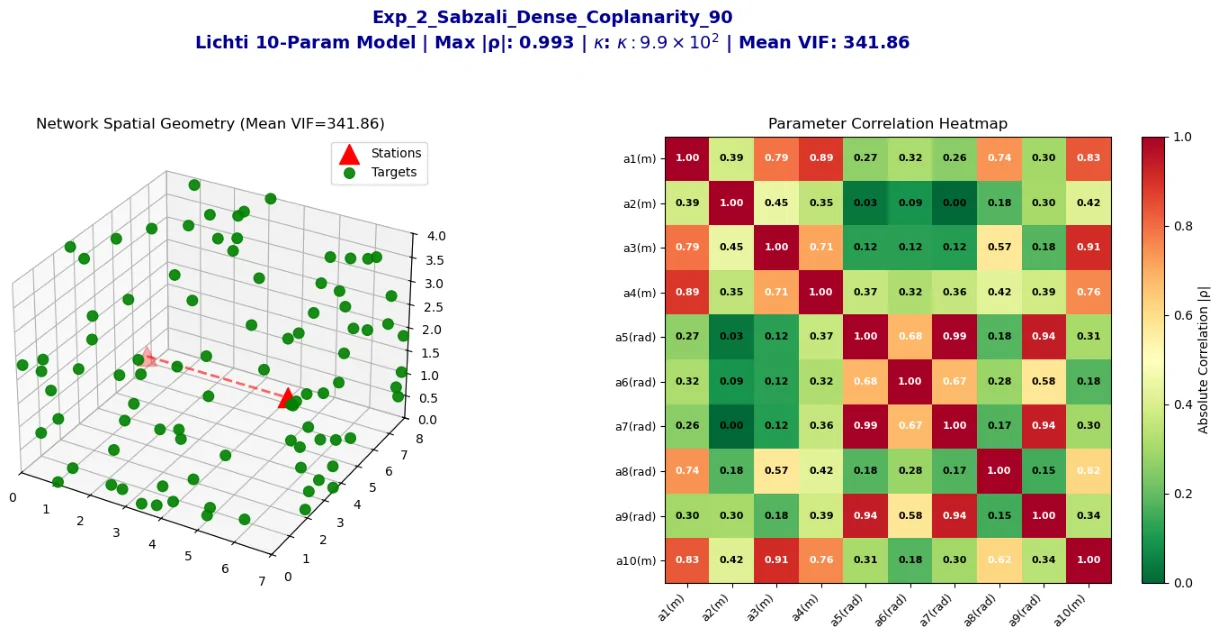
Spatial distribution of targets and parameter correlation heatmap for Exp 2 (Sabzali dense coplanarity constraint with 90 points and equielevational dual-station scanning).

Exp 2 reproduces the dense coplanarity constraint framework. As shown in Figure 2, although 90 spherical targets cover the walls, the heatmap on the right ruthlessly reveals that max|ρ| climbs to 0.99. More fatally, under the accumulation of parameter redundancy, the system Mean VIF skyrockets to 342, with variance experiencing catastrophic inflation. This experiment powerfully proves that violently stacking target numbers not only substantially increases the economic and time costs of field measurement, but also cannot break the angular coupling dead angle shrouded by the zenith-edge paradox.

### 5.2. Geometric Collapse and Distortion of Random Sparse Networks

**Figure 3:**
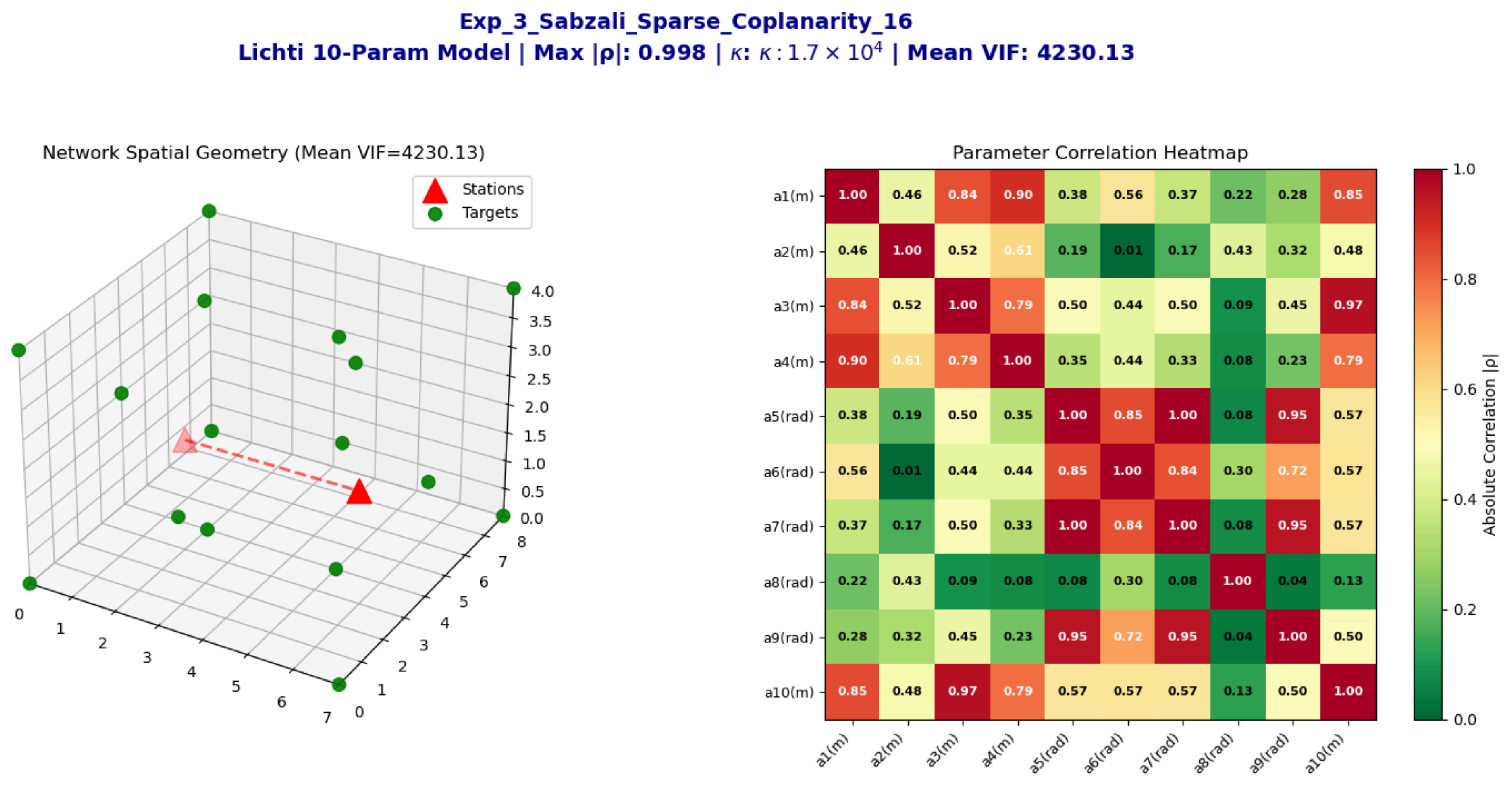
Spatial distribution of targets and parameter correlation heatmap for Exp 3 (Sabzali sparse coplanarity constraint with 16 points and equielevational dual-station scanning).

When spherical targets in the coplanarity constraint model are crudely reduced to 16 without scientific guidance (Exp 3), the sparse and random distribution (left side of Figure 3) directly triggers the complete collapse of the normal equations. As shown in Table 1, its matrix condition number *k* soars to 16,800, Mean VIF breaks through 4230, the heatmap presents large areas of near-singular dead zones (max|ρ|=1.0), and CPs fitting accuracy completely fails (error increases by an order of magnitude). This confirms from the reverse direction that in networks lacking geometric tension evaluation and design, once rigid epipolar constraints lose the support of key spatial corners, the floating relative network is highly prone to severe geometric model distortion and topological drift.

### 5.3. Parameter Decoupling of VIF Dimensionless Optimized Configuration

**Figure 4:**
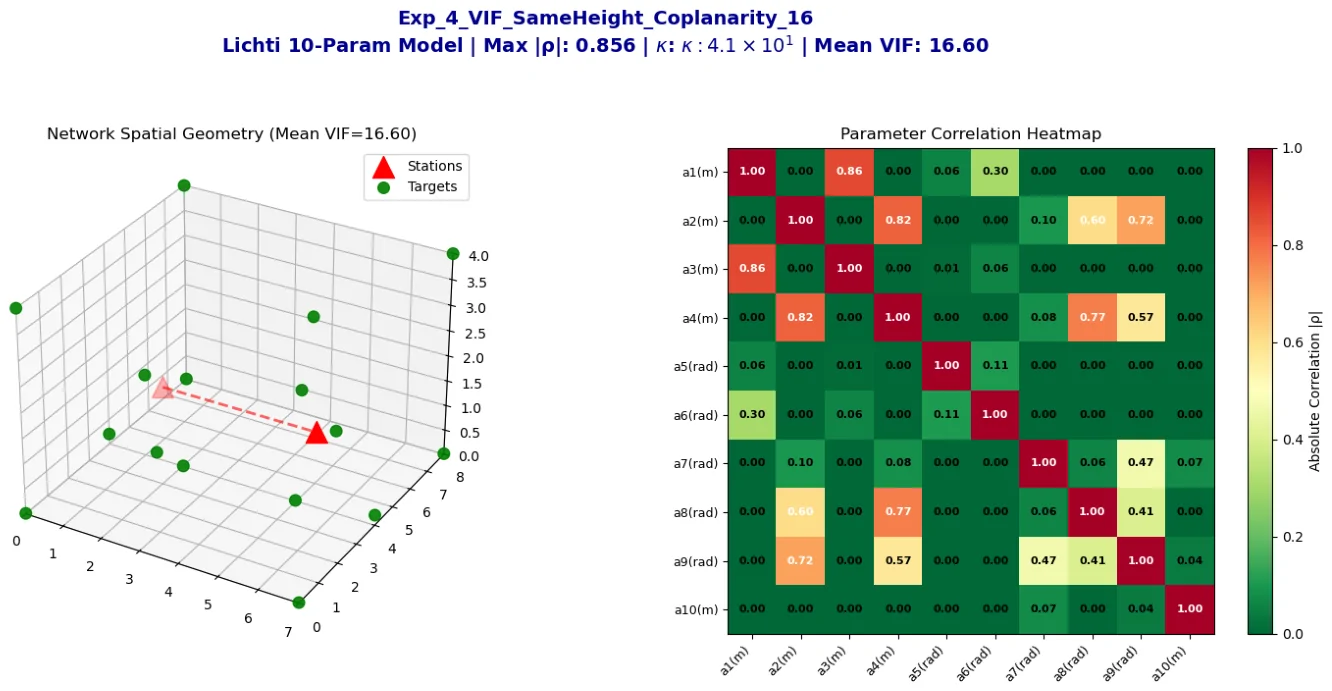
Spatial distribution of targets and parameter correlation heatmap for Exp 4 (VIF-optimized network with coplanarity constraint, 16 points and equielevational dual-station scanning).

Exp 4 introduces for the first time 16 spherical targets meticulously selected by the differential evolution algorithm under dimensionless VIF driving. As shown in Figure 4, the optimization algorithm precisely and actively allocates target points to the eight extreme corner points of the spatial bounding box, and forcibly places a “zenith decoupling anchor point” directly above the instrument. This extremely exquisite layout causes the originally dark high-risk coupling regions in the heatmap to largely dissipate, with the maximum correlation coefficient dropping to 0.86. Meanwhile, Table 1 data show that the condition number *k* plummets to the well-conditioned 41, and the system Mean VIF drops to 17, with variance inflation fundamentally curbed. This proves that the VIF tensor not only fully unleashes the mathematical potential of coplanar physical equations, but also avoids unnecessary diminishing marginal returns.

### 5.4. Absolute Lower Bound of Parameter Correlation

**Figure 5:**
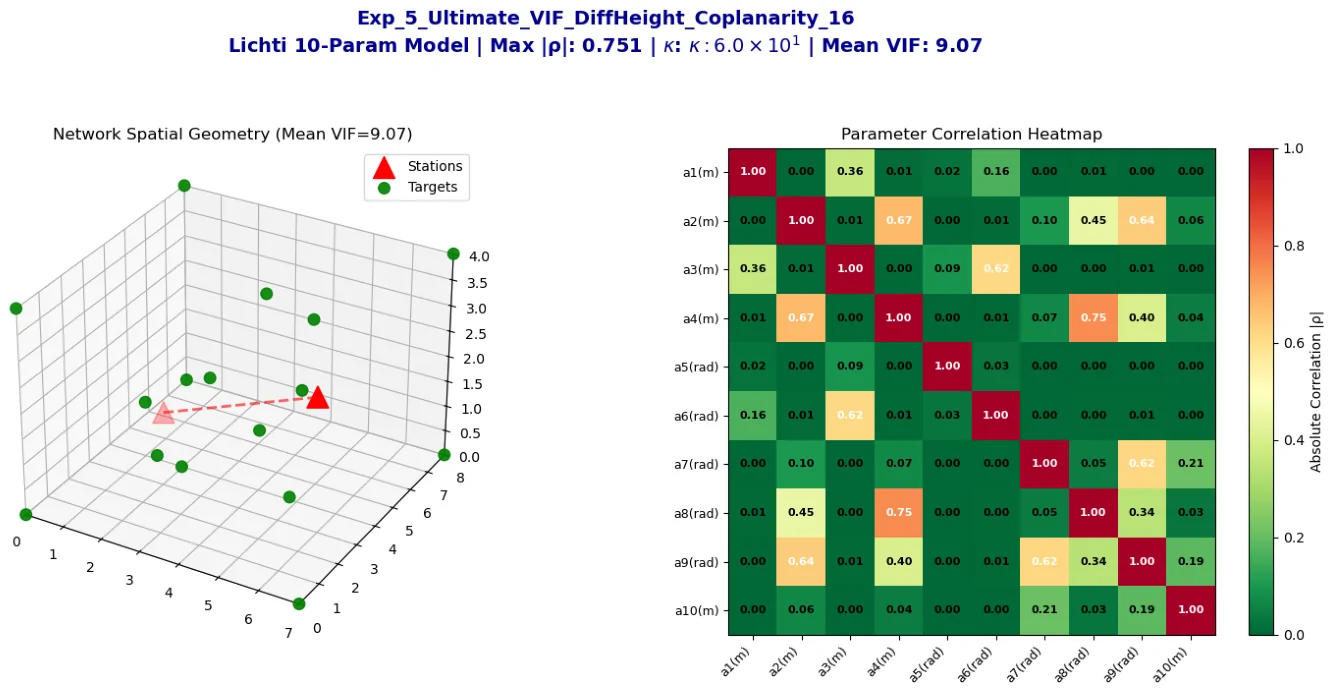
Spatial distribution of targets and parameter correlation heatmap for Exp 5 (VIF ultimate scheme with coplanarity constraint, 16 points and dual-station height-difference scanning).

In Exp 4, although parameter decoupling is remarkably effective, limited by the symmetry hazard of the azimuth baseline caused by setting the two scanning stations at the same elevation, the maximum parameter correlation coefficient ρ remains at 0.85. To thoroughly extract the limit performance of the adjustment equations, Exp 5 precisely implements physical intervention on the excellent VIF geometric configuration: artificially introducing an instrument setup height difference of ΔZ=1.2 m (the scanning station elevation difference is clearly visible on the left side of Figure 5). The ultimate adjustment results present a light-colored low-correlation distribution (right side of Figure 5). Not only does the condition number stabilize at the excellent level of 60, but the a posteriori variance factor ${\hat{\sigma }}_{0}^{2}\approx 1.19$ proves that the residual field is as pure as white noise. The core breakthrough is that the system Mean VIF is forcibly suppressed to 9 (breaking through the ideal defense line of 10), and the maximum correlation coefficient max|ρ| is firmly locked at 0.75. This value is by no means an accidental compromise due to insufficient optimizer computational power, but the hard mapping of the absolute mathematical physical limit. In the NIST 10-parameter model, core range errors are jointly controlled by $a_{3}\sin V+a_{10}$. Due to the hardware structure of TLS instruments, the zenith angle detection field of view is necessarily limited (e.g., $V\in [0^{{}^{\circ}},150^{{}^{\circ}}]$); therefore, the partial derivative sinV≥0 always holds. According to the Pearson correlation coefficient theorem in statistics between a constant-term column vector ($a_{10}$) and a non-negative variable column vector (sinV), there exists a theoretical lower bound strictly constrained by the Cauchy–Schwarz inequality:

(9) $$\rho_{min}=-\frac{\mathrm{Mean}(\sin V)}{\sqrt{\mathrm{Mean}(\sin^{2}V)}}$$

Under the most ideal limit bipolar distribution (data concentrated at zenith and azimuth), this theoretical limit converges to $-1/\sqrt{2}\approx -0.707$. The observation domain accuracy of Exp 5 not only numerically reaches dr=0.27 mm, but its solved ρ=0.75 achieves approximation to the statistical physical limit (0.707), representing near-optimal parameter decoupling within the instrument’s field-of-view constraints [33,34].

### 5.5. Real-World Validation Using Faro Focus 350 Measured Data

**Figure 6:**
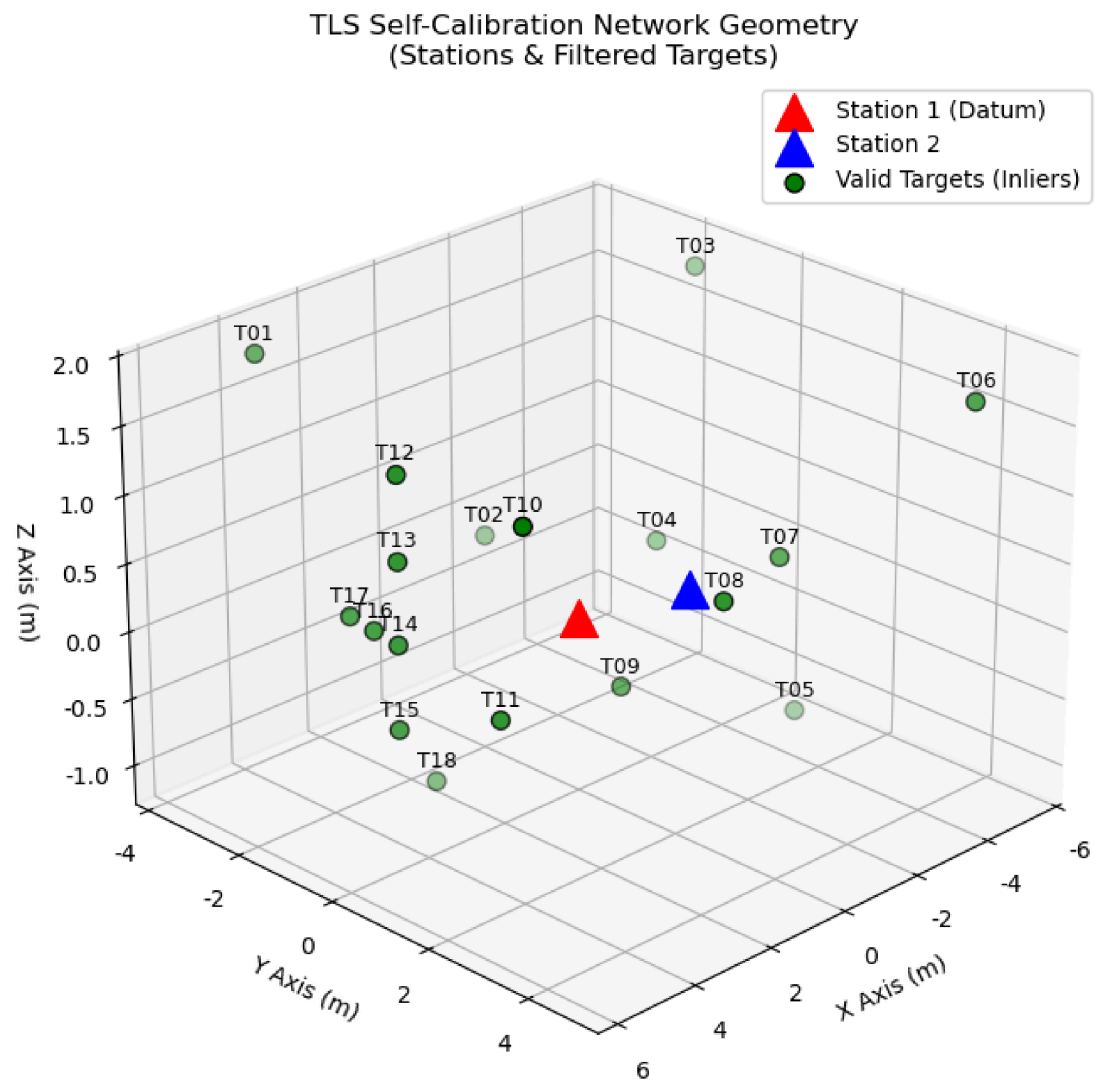
Three-dimensional geometric distribution map of 18 targets in the Faro Focus 350 physical calibration field.

Based on the Faro Focus 350 calibration field constructed in Section 4, we deployed the final 18 spherical targets in physical space according to the three-dimensional spatial topology configuration output by the VIF algorithm (as shown in Figure 6), and forcibly implemented high-low setup physical intervention.

After compensation through 10-parameter coplanarity-constrained adjustment, the system’s range residual RMS (dr) steadily converges to approximately 1.05 mm, and the azimuth angle (dh) and zenith angle (dv) residual RMS, respectively, converge to 18.2″ and 22.4″. This set of posterior precision indicators not only fully complies with the hardware nominal spherical coordinate observation bottom noise limit of the Faro Focus 350, but more fundamentally proves that even with the target number reduced to 18, because the VIF network provides extremely strong geometric rigid tensors, the adjustment model can still perfectly absorb the internal systematic distortion of the instrument, causing the residual field to successfully degrade into pure white noise conforming to Gaussian distribution. A limitation of the current field validation is its reliance on a single scanner model and a dual-station configuration. Future studies should test this optimization framework across various scanner architectures (e.g., Leica, Trimble) to assess robustness and multi-station sensitivity.

## 6. Conclusions

This paper targets the industrial pain point of collapsed field efficiency in high-precision in situ self-calibration of terrestrial laser scanners. By introducing the dimensionless VIF for multi-dimensional spatial minimal optimization, and deeply integrating the NIST micro 10-parameter physical model with the coplanarity-constrained collinearity equation system, an innovative network design theoretical derivation is completed.

The VIF-driven minimal 16-target optimization scheme, supplemented by dual-station big height difference physical intervention, optimizes the normal equation condition number to an extremely low, well-conditioned level. The system approaches the theoretical statistical lower bound, challenging the traditional cognition that high-precision calibration must rely on massive targets.

As three-dimensional digitization evolves toward ubiquitous intelligent perception, the minimal geometric functional model established by this scheme possesses broad potential application prospects. Future research could hypothetically embed this dimensionality reduction system into the tightly coupled adjustment framework of TLS and high-frequency Inertial Measurement Units (IMUs), achieving target-free continuous online dynamic self-calibration based on Simultaneous Localization and Mapping (SLAM) loop closure; simultaneously, for low-cost solid-state Micro-Electromechanical Systems (MEMS) LiDAR with stringent assembly tolerances, this minimal calibration paradigm could provide value for their array verification, and online calibration [35].

## Figures and Tables

**Table 1:** Performance and precision results of adjustment networks for each group.

Group	max|ρ|	Condition Number *k*	Mean VIF	Raw CPs RMS	${\hat{\sigma }}_{0}^{2}$	*dr*/mm	*dh*/″	*dv*/″
Exp1	0.91	548	41	$7.51\times 10^{-4}$	1.06	0.36	13.6	47.9
Exp2	0.99	989	342	$2.47\times 10^{-4}$	1.16	0.36	4.7	36.1
Exp3	0.99	16,800	4230	$3.25\times 10^{-3}$	1.08	1.19	103.4	232.4
Exp4	0.86	41	17	$1.84\times 10^{-4}$	1.14	0.52	22.2	18.0
Exp5	0.75	60	9	$1.31\times 10^{-4}$	1.19	0.27	8.7	26.4

## Data Availability

The original contributions presented in this study are included in the article. Further inquiries can be directed to the corresponding author.

## Appendix A

**Table A1:** Physical meaning explanation of the NIST 10-parameter core backbone model.

Parameter	Physical Description	Error Category
$a_{1}$	Horizontal beam offset	Beam and axis offset
$a_{2}$	Vertical beam offset	Beam and axis offset
$a_{3}$	Trunnion axis offset	Beam and axis offset
$a_{4}$	Mirror offset	Mirror error
$a_{5}$	Vertical circle index error	Axis tilt and alignment error
$a_{6}$	Horizontal beam tilt component	Axis tilt and alignment error
$a_{7}$	Vertical beam tilt component	Axis tilt and alignment error
$a_{8}$	Mirror tilt	Mirror error
$a_{9}$	Trunnion axis tilt/non-orthogonality error	Axis tilt and alignment error
$a_{10}$	Range zero offset error	Beam and axis offset
